# Will there be a rapid change towards an EVT-only paradigm?

**DOI:** 10.1177/15910199211011880

**Published:** 2021-04-22

**Authors:** Mayank Goyal, Johanna M Ospel, Michael D Hill

**Affiliations:** 1 Calgary Stroke Program, Department of Clinical Neurosciences, University of Calgary, Calgary, Canada; 2 Department of Radiology, University Hospital Basel, University of Basel, Basel, Switzerland; 3 Department of Radiology, University of Calgary, Calgary, Canada

In this letter, we comment on the results of the SKIP^1^ and DEVT trials,^2^ and the recently presented MR CLEAN NO IV trial, which we have read with great interest. Although the results of these trials leave room for interpretation and may not be sufficient yet to change practice because the non-inferiority margins in the SKIP trial and the MR CLEAN NO IV trial (presented on March 18 at the international stroke conference) were not met, they may suggest that a so-called “dry” endovascular treatment (EVT) approach could be non-inferior compared to EVT with concurrent intravenous alteplase. Together with the recently published DIRECT-MT trial^3^ that showed non-inferior outcomes with EVT-only treatment, they suggest that intravenous alteplase, which has been a cornerstone of acute ischemic stroke treatment, might not add substantial value in large vessel occlusion stroke when mechanical recanalization of the occluded blood vessel can be achieved quickly. If similar ongoing trials (SWIFT-DIRECT, NCT03192332; DIRECT-SAFE, NCT03494920) will confirm these results, it seems likely that there will be sufficient evidence to change clinical practice. What would be the implications of such a paradigm change in large vessel occlusion stroke treatment? First, foregoing intravenous alteplase in large vessel occlusion stroke patients can improve treatment speed due to faster decision-making and workflow times because time-consuming initial admissions to a primary stroke center for IV thrombolysis followed by inter-hospital transfer to a comprehensive stroke center for EVT (“drip-and-ship”) can be avoided.^4^ Second, alteplase is expensive - the costs for a single dose in the United States runs into thousands of dollars. Thus, an EVT-only strategy will save costs. Third, an EVT-only treatment paradigm would allow alternate treatments that should not be used with thrombolysis, such as the cytoprotective agent nerinetide. In the ESCAPE-NA1 trial,^5^ a clear improvement in clinical outcomes with nerinetide was observed in large vessel occlusion stroke patients treated with EVT if they did not receive intravenous alteplase. In those treated with alteplase, no benefit was seen, due to a pharmacological interaction which resulted in cleavage of nerinetide by alteplase breakdown products. The currently ongoing ESCAPE-NEXT trial (NCT04462536) seeks to confirm these preliminary results. Individually, the SKIP and DEVT trials and similar studies may not have reached their primary end-point or may not have had a sufficiently narrow non-inferiority boundary to change guidelines. But given the possible implications above, we believe that EVT as a stand-alone treatment could soon become the favoured approach in large vessel occlusion patients in situations in which EVT can be initiated quickly. It is likely that alternative thrombolytic agents such as tenecteplase will also influence whether an EVT-only paradigm will further prevail. Additionally, with improved understanding of clot characteristics and analysis of sub-groups from large EVT trials, many centers may choose to tailor their triage and treatment decisions by selectively adopting a direct-to-EVT paradigm in certain patient sub-groups, such as patients with tandem occlusion or those with large clot burden, until more definitive data become available.
